# Non-invasive brain stimulation for posttraumatic stress disorder: a systematic review and meta-analysis

**DOI:** 10.1038/s41398-020-0851-5

**Published:** 2020-05-28

**Authors:** Rebecca L. D. Kan, Bella B. B. Zhang, Jack J. Q. Zhang, Georg S. Kranz

**Affiliations:** 1 Department of Rehabilitation Sciences, The Hong Kong Polytechnic University, Hong Kong, SAR China; 2 Department of Psychiatry and Psychotherapy, Medical University of Vienna, Vienna, Austria; 3 The State Key Laboratory of Brain and Cognitive Sciences, The University of Hong Kong, Hong Kong, SAR China

**Keywords:** Psychiatric disorders, Neuroscience, Psychiatric disorders, Neuroscience

## Abstract

Approximately 7–9% of people develop posttraumatic stress disorder in their lifetime, but standard pharmacological treatment or psychotherapy shows a considerable individual variation in their effectiveness. Repetitive transcranial magnetic stimulation (rTMS) and transcranial direct current stimulation (tDCS) hold promise for the treatment of posttraumatic stress disorder. The objective of this meta-analysis was to summarize the existing evidence on the therapeutic effects of these brain stimulation treatments on posttraumatic core symptoms. We systematically retrieved articles published between 1st January 2000 and 1st January 2020 comparing the effects of active with sham stimulation or no intervention in posttraumatic patients from eight databases. Random-effects model was used for meta-analysis. Meta-regression and subgroup meta-analysis was performed to investigate the influence of stimulation dose and different stimulation protocols, respectively. 20 studies were included in this review, where of 11 randomized controlled trials were subjected to quantitative analysis. Active stimulation demonstrated significant reductions of core posttraumatic symptoms with a large effect size (Hedge’s *g* = −0.975). Subgroup analysis showed that both excitatory and inhibitory rTMS of the right dorsolateral prefrontal cortex led to symptom reductions with a large (Hedges’ *g* = −1.161, 95% CI, −1.823 to −0.499; *p* = 0.015) and medium effect size (Hedges’ *g* = −0.680, 95% CI: −0.139 to −0.322; *p* ≤ 0.001) respectively. Results further indicated significant durability of symptom-reducing effects of treatments during a two to four weeks period post stimulation (Hedges’ *g* = −0.909, 95% CI: −1.611 to −0.207; *p* = 0.011). rTMS of the right dorsolateral prefrontal cortex appears to have a positive effect in reducing core symptoms in patients with posttraumatic stress disorder.

## Introduction

Posttraumatic stress disorder (PTSD) is a common psychiatric disorder that occurs after direct or indirect exposure to a traumatic event. PTSD is characterized by four core symptoms including re-experiencing, hyperarousal, avoidance of trauma-related stimuli and negative cognition and mood^1^. Approximately 7 to 9% of people develop PTSD in their lifetime, whereas the rate is estimated to be much higher in military veterans^2,3^. PTSD is frequently associated with mood dysregulation, addiction, shame, feelings of guilt, aggression, shallow sleep and poor physical health, thereby leading to occupational disability and poor quality of life^4^. Furthermore, over half of patients with PTSD also suffer from a major depressive disorder (MDD)^5,6^. However, standard pharmacological treatment or psychotherapy has only been partly successful, showing a considerable individual variation in their effectiveness^7^. Hence, various studies have been conducted to explore alternative treatments. Here, non-invasive brain stimulation (NIBS) including repetitive transcranial magnetic stimulation (rTMS) and transcranial direct current stimulation (tDCS) received much attention lately, given their ability to modulate cortical excitability. Indeed, research employing animal models and neuroimaging studies in humans suggest that altered brain excitability could be a major pathophysiological factor contributing to PTSD. A hyperactivity of the amygdala and dorsal anterior cingulate cortex, regions that are known to promote fear responses in animals and humans, has been associated with PTSD. On the other hand, hypoactivity of the ventromedial and dorsolateral prefrontal cortex (VMPFC and DLPFC, respectively) has been reported, regions that are known to be involved in the suppression of fear responses^8–11^. Specifically, the right hemisphere’s dominant role in stress modulation has been linked to PTSD, with studies indicating structural abnormalities especially of the right hemisphere^12^.

rTMS and tDCS are frequently employed as save alternative options to pharmacotherapy for the treatment of a number of psychiatric disorders. The magnetic field elicited by rTMS passes through the scalp and skull and changes cortical and subcortical activity in specific brain networks without injury. In general, high frequency (HF) stimulation (>5 Hz) increases cortical excitability, while low frequency (LF) stimulation (≤1 Hz) decreases cortical excitability^13^. In addition, a patterned form of rTMS called theta-burst stimulation (TBS) was established in 2005^14^. Standard protocols of TBS consist of 50 Hz bursts of 3 pulses that are repeated at 5 Hz to reach a total number of 600 pulses. TBS can be applied continuously (cTBS) or in an intermittent form (iTBS), while the latter exhibits facilitatory, and the former inhibitory effects on neural excitability^14^. tDCS leads to sub-threshold shifts of resting membrane potentials by applying direct currents via scalp electrodes over targeted cortical areas^15^. Anodal tDCS increases the excitability of cortex whereas cathodal tDCS decreases it. Several meta-analyses on the effects of rTMS and tDCS in depression indicate that presumed brain excitability changes of these two NIBS techniques may effectively reduce depressive symptoms^16,17^. In addition, a number of studies also explored the potential of rTMS and tDCS in the treatment of PTSD in order to increase the inhibitory control of amygdala activity. Four reviews and meta-analyses investigating the effects of rTMS in PTSD have been published so far. One review and one meta-analysis indicated promising effects of rTMS on PTSD symptom reductions. However, the results remain preliminary, as these two studies only included three and five randomized controlled trials (RCTs), respectively^18,19^. A more recent meta-analysis including nine original studies (six RCTs) demonstrated positive effects of rTMS on PTSD with an effect size of −0.88^20^. Another meta-analysis including 11 RCTs suggested that LF rTMS could reduce overall PTSD and depression symptoms^21^. In addition, several studies also applied tDCS on the DLPFC in order to alleviate mood symptoms in people with PTSD with promising effects^22,23^. However, no meta-analysis has been conducted so far to summarize these effects. Therefore, there is still limited meta-analytic research investigating different rTMS and tDCS protocols on core symptoms of PTSD, as well as the relationship between stimulation parameters and effect sizes. Thus, the aim of the current study was to (1) summarize existing evidence on the therapeutic effects of rTMS and tDCS on core symptoms of PTSD using meta-analysis and to (2) probe the association between different stimulation parameters and effect sizes using meta-regression.

## Methods

### Data source and literature search

The present review followed the Preferred Reporting Items for Systematic Review and Meta-Analysis (PRISMA)^24^. Four English bibliographic databases including PubMed, PsycINFO, Web of Science and EMBASE and four Chinese databases including the Chinese National Knowledge Infrastructure (CNKI), WeiPu, WanFang and the Chinese Biomedical Literature Database (CBM), were systematically searched for articles published from 01 January 2000 to 01 January 2020 using the key words “Repetitive Transcranial Magnetic Stimulation” OR “rTMS” OR “Theta-burst stimulation” OR “TBS” OR “Transcranial direct current stimulation” OR “tDCS”) AND (“Posttraumatic stress disorder” OR “PTSD”). In addition, we manually screened reference lists of related published reviews and meta-analyses for additional relevant studies. Two authors (RLDK and BBBZ) independently identified potential studies by reading the title and abstract, and any disagreement was settled through discussion with the third author (JJQZ).

### Inclusion and exclusion criteria

We included RCTs published in English and Chinese language with patients having a diagnosis of PTSD according to standard operationalized diagnostic criteria. We included studies comparing any form of rTMS or patterned TMS or tDCS with sham stimulation or no intervention in the treatment of PTSD. We excluded studies published as conference abstracts without full text, as well as book chapters and dissertations. Poor quality RCTs (PEDro<6) were also excluded^25^.

### Quality assessment and data extraction

After identifying potentially eligible studies, full texts were retrieved and two authors (RLDK and BBBZ) extracted the relevant information and assessed the quality of each article independently. Any disagreements were resolved by consultation with the third author (JJQZ). The Physiotherapy Evidence Database (PEDro) Scale was used to assess the quality of included RCTs. Extracted information included: the study design; diagnosis and group membership of participants; stimulation details; main outcomes and assessment time points.

### Statistical analysis

All statistical analyses were conducted using the software package Comprehensive Meta-analysis version 3.0 for Windows. For articles reporting incomplete data, corresponding authors were contacted by email. The formula SD = SEM × √*n* (*n* = sample size) was used for conversion of standard errors of the mean (SEM) into standard deviations (SD). GetData Graph digitizer 2.26 (http://www.webcitation.org/77dui8IFb) was used to extract data that were reported as a graph only^26^.

#### Individual study effect estimates

PTSD symptoms in individual trials were measured using standardized rating scales, including the Clinician-Administered PTSD Scale (CAPS), an observer-rating scale, and the self-report scale PTSD Checklist (PCL)^27,28^. High convergent validity of the CAPS and PCL scales has been demonstrated^29^. The individual effect sizes were estimated using absolute change scores (i.e., post- minus pre-stimulation scores) to correct for baseline differences between groups. The standardized mean difference (Hedges’ *g*) and 95% confidence intervals (CIs) comparing subjects with and without NIBS was computed for each trial. Hedges’ *g* is a variation of Cohen’s d, which corrects for possible bias of small sample sizes^30^.

#### Summary effect estimates

Random-effects meta-analysis was performed given the clinical and methodological diversity among included trials. Heterogeneity among the included studies was assessed using Higgins’I^2^ statistic^31,32^. Meta-regression was used to test the relationship between protocol type, dose and effect size. Subgroup analysis was used to explore the effects of different rTMS protocols (i.e. targets, frequencies, TMS as monotherapy or as augmentation treatment) on PTSD symptom reductions. Sensitivity analysis was performed using the leave-one-out method in case of significant results. Publication bias was assessed by visual inspection (funnel plot) and Egger’s test in case of more than ten articles^33,34^. The statistical threshold was set at *p* < 0.05 and *p* < 0.1 (two-tailed) for the main tests and for the Egger’s test, respectively.

## Results

### Literature search results and characteristics of included studies

**Fig. 1:**
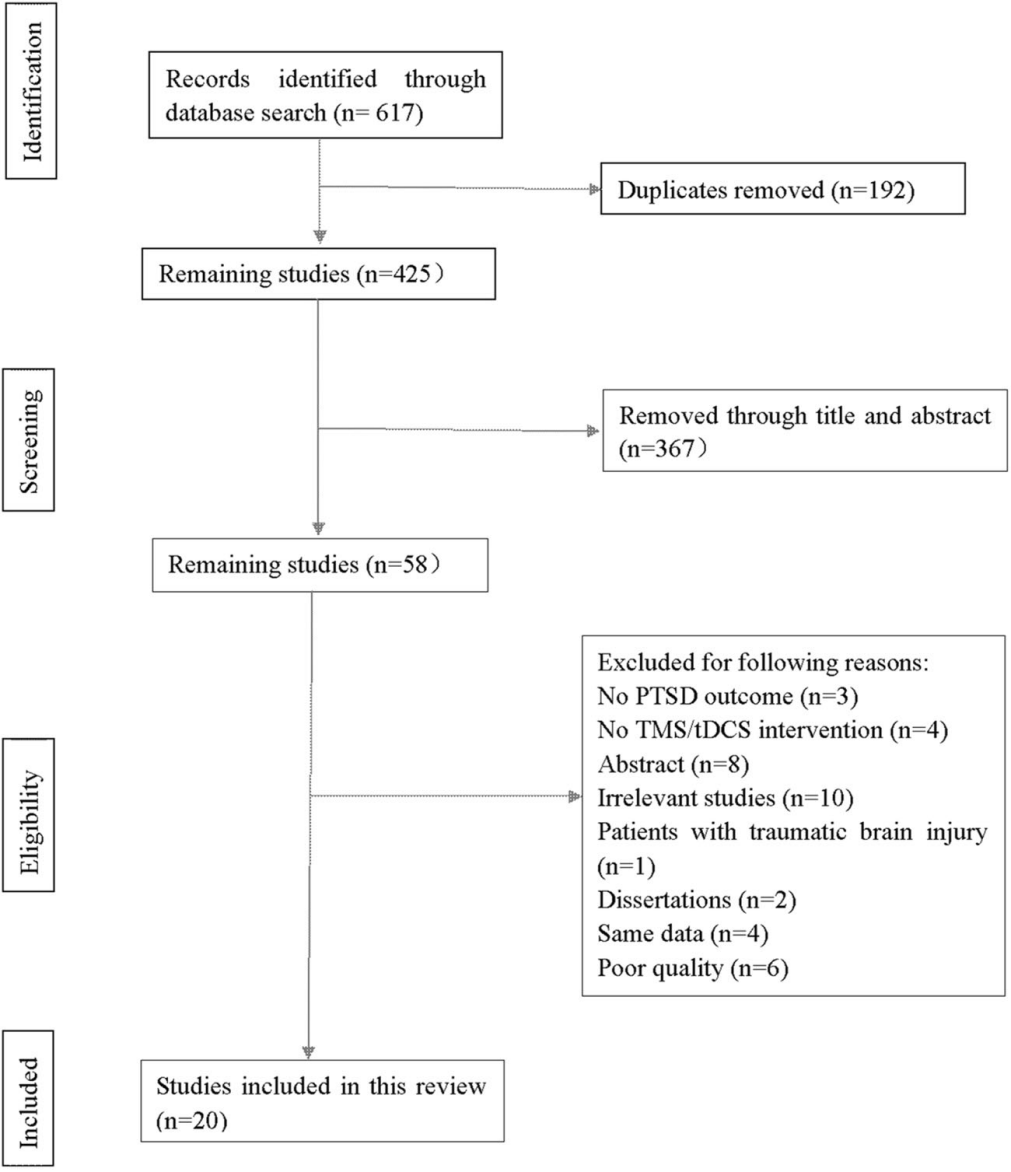
PRISMA flowchart. Process of literature search.

Our search strategy yielded 617 results in total. After the removal of duplicates, 425 articles were identified, whereas 58 studies remained after reading titles and abstracts. Studies were further excluded because they were irrelevant to the topic (ten studies), they were only published as abstracts (eight studies), they did not include rTMS or tDCS as intervention (four studies), they did not report PTSD symptoms as outcome measure (three studies), they included participants with traumatic brain injury (one study), they were published as dissertations (two studies), or they included the same data (four studies). Moreover, six studies were excluded given their poor quality (a PEDro score < 6). The remaining 20 studies were included in the present review. 15 of them were RCTs while five were single group studies and were therefore not included in the quantitative analysis (see study flow chart in Fig. 1).

Table 1 shows the characteristics of included studies. Nine studies investigated PTSD symptoms in veterans^23,26,35–41^. Seven studies reported comorbidity with MDD in all subjects^8,26,39–43^, while other studies mentioned comorbid anxiety or panic in some subjects. Five studies used a minimum PCL score as inclusion criterion, ranging from 33 to 50^22,35,38,41,44^. Stimulation intensity of rTMS studies varied between 80% and 120% of the resting motor threshold (RMT). In addition, two studies investigated tDCS as intervention^22,23^.

**Table 1:** Study characteristics of included studies.

Study	Study design	Subjects	Group location	Stimulation type	Target	Total dose	Intensity	Pulse per session	Sham method	Main outcomes	Assessment time
Ahmadizadeh et al.^35^	RCT	PTSD	B-rTMS = 19 R-rTMS = 19 Sham = 20	rTMS/20 Hz	RDLPFC and BDLPFC	10 sessions 24000 pulses	100% RMT	1200	Sham coil	PCL-M	Baseline Session5 Session 10
Boggio et al.^45^	RCT	PTSD	RDLPFC = 10 LDLPFC = 10 Sham = 10	rTMS/20 Hz	RDLPFC and LDLPFC	10 sessions 16000 pulses	80%RMT	1600	Sham coil	PCL-5 Treatment Outcome PTSD Scale HARS HDRS	Baseline Session5 Session 10 2 weeks FU 4 weeks FU 8 weeks FU 12 weeks FU
Carpenter et al.^42^	Open-label	PTSD and MDD	*N* = 35	rTMS/5 Hz	LPFC	40 sessions 3000 pulses	120%RMT	3000	N/A	PCL-5 IDS-SR PHQ-9 DASS PSS CGI-S PGI-S	Pre Post
Cohen et al.^46^	RCT	PTSD	10Hz-rTMS = 10 1Hz-rTMS = 8 sham rTMS = 6	rTMS/1 Hz and 10 Hz	RDLPFC	10 sessions 200 min	80% RMT	/	Coil rotation (vertical to RDLPFC)	PCL-5 Treatment Outcome PTSD Scale HARS HDRS CAPS	Baseline 5 session 10 session 2 weeks FU 14 days
Fryml et al.^36^	RCT	PTSD	EG = 5 CG = 3	rTMS/10 Hz	RDLPFC + LDLPFC	8 sessions 48000 pulses	120% RMT	6000	Sham stimulation	CAPS Ham-D Ham-A PTSD Checklist	Pre post
Isserles et al.^47^	RCT	PTSD	9:9:8	dTMS/20 Hz	Bilateral MPFC	12 sessions 20160 pulses	120% RMT	1680	Sham coil	CAPS PSS-SR HDRS-24 BDI	Pre post 2 weeks FU 2 months FU
Kozel et al.^37^	RCT	PTSD	EG (rTMS+CPT) = 54 CG (sham rTMS + CPT) = 49	rTMS/1 Hz	RDLPFC	12 sessions 21600 pulses	110% RMT	1800	Sham coil	CAPS PCL M-PTSD QIDS-SR	Baseline Session 5 Session 9 1 month FU 3 months FU 6 months FU
Kozel et al.^38^	RCT	PTSD	1Hz-rTMS =17 10Hz-rTMS = 18	rTMS/1 Hz and 10 Hz	RDLPFC	36 sessions 86400 pulses	110% RMT	2400	/	PCL-5 CAPS QIDS-SR MADRS Pain Score NSI	Pre Post 1 month FU 3 months FU
Nam et al.^49^	RCT	PTSD	EG = 7 CG = 9	rTMS/1 Hz	RDLPFC	15 sessions 18600 pulses	100%MT	1200	Coil rotation (vertical to RDLPFC)	CAPS	Pre Session 10 1 week FU 5 weeks FU
Osuch et al.^50^	RCT (cross over)	PTSD	Active rTMS = 5 Sham rTMS = 5	rTMS/1 Hz	RDLPFC	20 sessions 36000 pulses	100%MT	1800	Coil rotation 45°to head)	CAPS HDRS IES	Pre Post
Oznur et al.^39^	Open-label	PTSD	*N* = 20	TMS/1 Hz	RDLPFC	6	80%	600	/	BDI BAI IES	/
Philip et al.^43^	Open-label	PTSD and MDD	*N* = 10	rTMS/5 Hz	LDLPFC	30 + 6 sessions 10800 pulses	120%RMT	3000	/	PCL QIDS	Pre Session 5 Session 10 Session 15 Session 20 Session 25 Post
Philip et al.^8^	Open-label	PTSD and MDD	*N* = 31	rTMS/5 Hz	LDLPFC	40	/	3000-4000	/	PCL IDS-SR MRI	Pre Post
Philip et al.^40^	RCT	PTSD and MDD	EG = 25 CG = 25	iTBS	RDLPFC	10 sessions 18000 pulses	80%AMT	1800	/	CAPS Social and Occupational Functioning Assessment Scale QOL PCL IDS-SR	Pre Post 1 month FU
Philip et al.^41^	RCT	PTSD and MDD	EG = 25 CG = 25	sTMS	/	10 sessions	/	/	/	PCL-5 PTSD threshold symptoms QIDS-SR	Pre Post
Rosenberg et al.^26^	RCT	PTSD and MDD	10 Hz = 6 1 Hz = 6	TMS/1 Hz and 5 Hz	LDLPFC	10 sessions	90%RMT	600	/	SCID-C Ham-D USC-REMT MSCS POMS	Pre Post 1 month FU 2 months FU
Watts et al.^44^	RCT	PTSD and MDD	EG = 10 CG = 10	rTMS/1 Hz	RDLPFC	10 sessions	90%RMT	/	Sham coil	CAPS PCL BDI STAI BNCE	Pre Post 1 month FU 2 months FU
Woodside et al.^48^	open-lable	PTSD and eating disorder	*N* = 14	rTMS (10 Hz and 20 Hz) and iTBS	DMPFC	20–30 sessions	120%RMT	3000 and 1500	/	PCL-C DERS	Pre Post
Ahmadizadeh et al.^22^	RCT	PTSD	EG = 18 CG = 16	Anodal tDCS	RDLPFC and LDLPFC	10 sessions	2 mA	/	/	PCL-5 BDI-II BAI	Pre Post 1 month FU
Wout et al.^23^	RCT	PTSD	EG = 6 CG = 6	tDCS	VMPFC	6 sessions	2 mA	/	/	SCR PCL-5	Pre Post 1 month FU

*RCT* randomized controlled trial, *B-rTMS* bilateral repetitive transcranial magnetic stimulation, *R-rTMS* right repetitive transcranial magnetic stimulation, *RDLPFC* right dorsolateral prefrontal cortex, *BDLPFC* bilateral dorsolateral prefrontal cortex, *RMT* resting moter threshold, *PCL-M* PTSD checklist military version, *LDLPFC* left dorsolateral prefrontal cortex, *PCL-5* PTSD checklist for DSM-5, *HARS* Hamilton Anxiety Rating Scale, *HDRS* Hamilton Depression Rating Scale, *FU* follow-up, *MDD* major depression disorder, *LPFC* left prefrontal cortex, *IDS-SR* Inventory of Depressive Symptomatology, *PHQ-9* Patient Health Questionnaire, *DASS* Depression Anxiety Stress Scale, *PS*: Perceived Stress Scale, *CGI-S* global illness severity, *PGI-S* patient self-rated version, *CAPS* Clinician-Administered PTSD Scale-II, *EG* experiment group, *CG* control group, *Ham-D* Hamilton Rating Scale for Depression, *Ham-A* Hamilton Rating Scale for Anxiety, *MPFC* medial prefrontal cortex, *PSS-SR* PTSD symptom scale-self report version, *HDRS-24* Hamilton Depression Rating Scale 24 items, *BDI* Beck Depression Inventory II, *CPT* cognitive processing therapy, *PCL* PTSD check list, *M-PTSD* Mississippi Scale for Combat Related PTSD, *QIDS-SR* quick inventory of depressive symptomatology-self report Version, *MADRS* Montgomery- Asberg Depression Rating Scale, *NSI* neurobehavioral symptom inventory, *BAI* The beck anxiety inventory, self-report, *IES* Impact of Events Scale, *MRI* magnetic resonance imaging, *QOL* quality of life, *SCID-C* Structured Clinical Interview for DSM-IV Axis I Disorders, Clinician Version, *USC-REMT* University of Southern California Repeatable Episodic Memory Test, *MSCS* Mississippi Scale of Combat Severity, *POMS* Profile of Mood States Subscales, *STAI* The State Trait Anxiety Inventory, *BNCE* The Brief Neurobehavioral Cognitive Examination, *DERS* Difficulties in Emotional Regulation Scale, *VMPFC* ventromedial prefrontal cortex, *BDI-II* The Beck depression inventory-II, *SCR* skin conductance reactivity.

### Quality assessment of included studies

The results of the quality assessment for the 15 RCTs are presented in the Supplementary Table S1. Eleven of them had a score of 8 on the PEDro scale, three studies had a score of 7 and one a score of 6.

### Meta-analysis results of RCTs

**Fig. 2:**
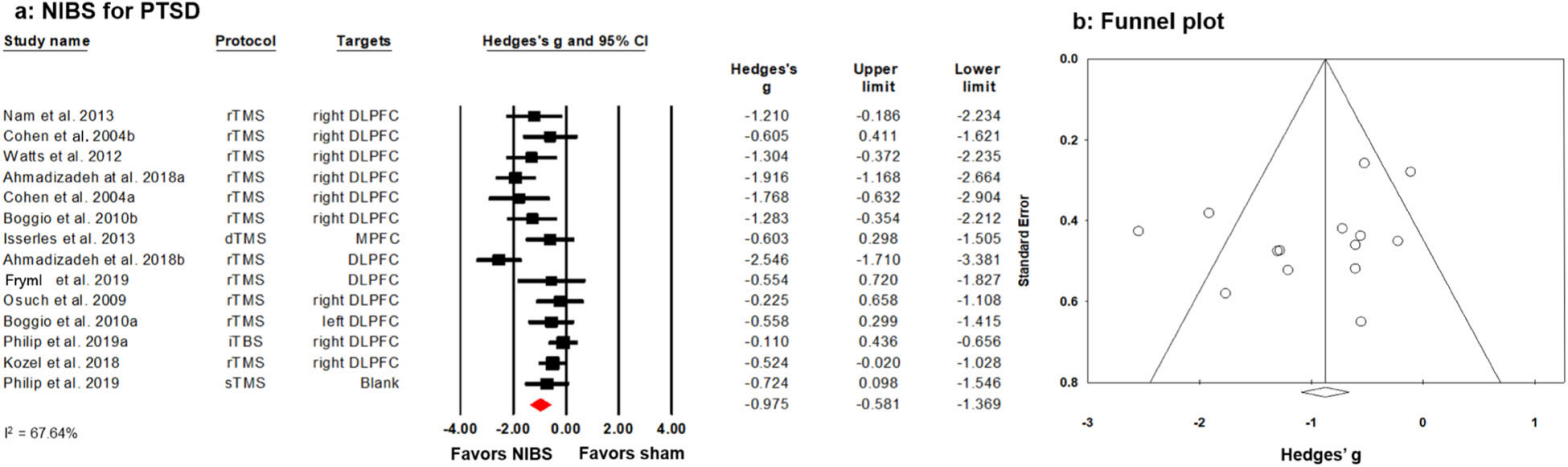
Effects of NIBS in PTSD. **a** Forest plot depicting studies comparing active with sham stimulation, summarizing to an effect size of −0.975. **b** The corresponding funnel plot comparing active with sham stimulation shows no publication bias; the Egger’s test is non-significant (*p* = 0.180).

Of the 15 RCTs (out of 20 studies included in our review), three RCTs included two separate subgroups^35,45,46^. However, two RCTs did not include a sham stimulation condition^26,38^, and studies investigating tDCS for PTSD were also excluded from the meta-analysis due to their low number (two studies) and large heterogeneity of the stimulation protocol^22,23^. Hence, 14 separate datasets of 11 RCTs including a total of 359 patients were subjected to cumulative meta-analysis. Results revealed that rTMS is an effective treatment to reduce core symptoms of PTSD with a large effect size (Hedges’ *g* = −0.975) and moderate heterogeneity of individual study estimates (*I*^2^ = 67.64%) (see Fig. 2a). The Funnel plot showed no publication bias (see Fig. 2b), which was confirmed by a non-significant Egger’s test (*p* = 0.180. However, meta-regression did not determine a significant dose effect as tapped by the number of sessions or total pulses. Furthermore, there were no significant differences between stimulation targets or stimulation frequencies on PTSD symptom reductions (all *p* > 0.05). Nevertheless, given the presumed variance on neural excitability, we performed exploratory post-hoc meta-analyses separately for target sites and stimulation frequencies.

#### Classification by protocol

##### Excitatory stimulation protocols

**Fig. 3:**
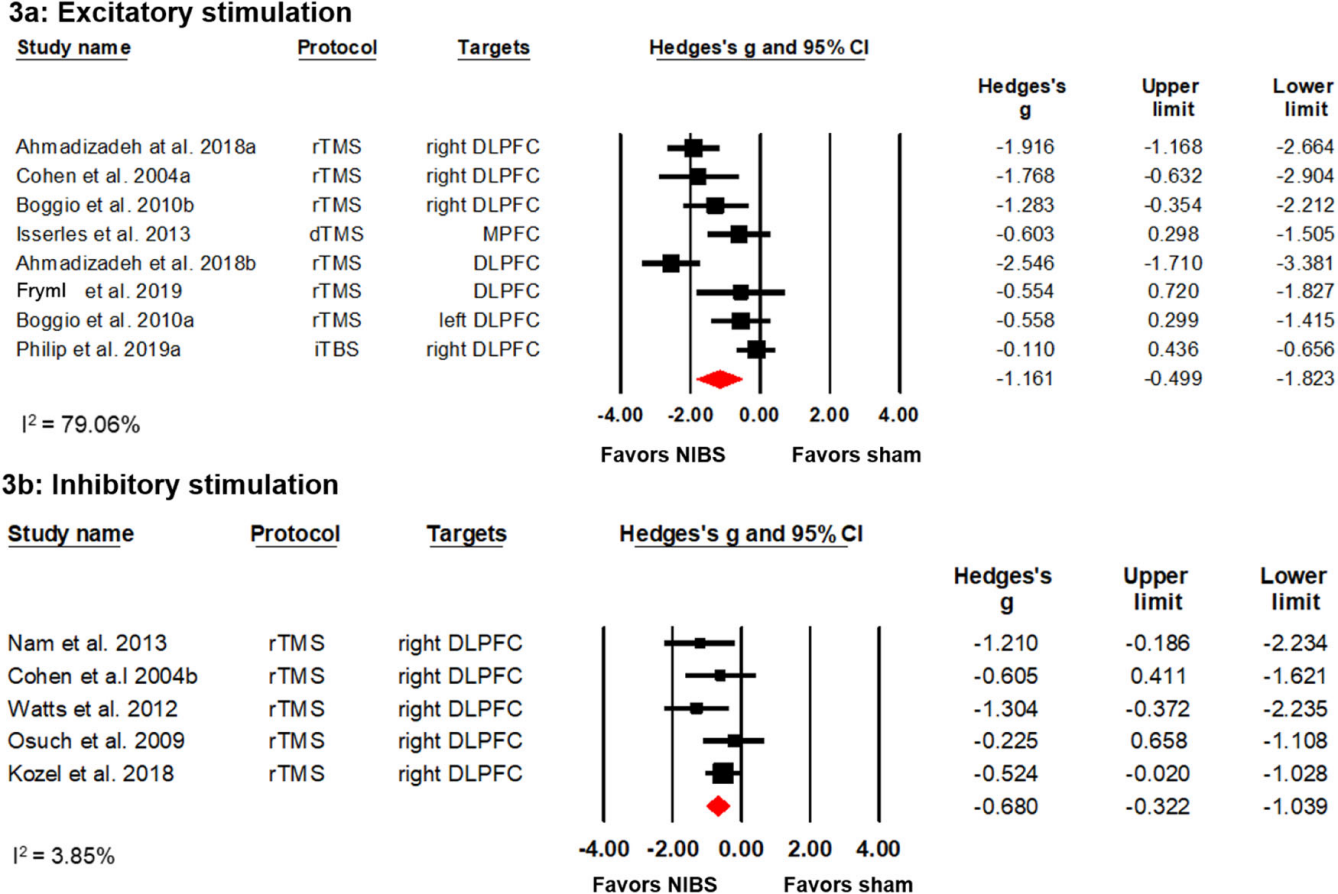
Effects of excitatory and inhibitory stimulation protocols in PTSD. **a** A forest plot showing studies that compared excitatory stimulation with sham stimulation. **b** A forest plot showing studies comparing inhibitory stimulation with sham stimulation.

Eight datasets (six studies) investigated the effects of excitatory stimulation on PTSD symptom reductions with 107 patients in the experimental and 103 patients in the control group. Six RCTs investigated HF rTMS^35,36,40,45–47^, whereas two studies explored the effects of dTMS and iTBS, respectively^40,47^. Despite a high heterogeneity of individual study estimates (*I*^2^ = 79.06%), the meta-analysis revealed a significant symptom reducing effect with a large effect size (Hedges’ *g* = −1.161) (see Fig. 3a). This result was robust to leave-one-out sensitivity analysis (Hedges’ *g* from −1.308 to −0.528).

Post-hoc analysis was conducted to investigate the effect of different stimulation targets. Four studies used HF rTMS of the right DLPFC^35,40,45,46^. In spite of a high heterogeneity of effect estimates (*I*^2^ = 83.32%), meta-analysis detected a significant positive effect with a large effect size (Hedges’ *g* = −1.225). Two studies applying high frequency stimulation on bilateral DLPFC found no significant positive effect. One study explored the effects of high frequency stimulation of the MPFC^47^ indicating no significant effect (*p* = 0.19), while one open-label study suggested that HF rTMS of the DLPFC may be effective to reduce PTSD symptoms^48^. Four studies explored the effects of excitatory rTMS on the left DLPFC^8,42,43,45^ but no meta-analysis was conducted since three of them were non-RCTs^8,42,43^. However, three of them showed a large^8,42,43^ and one a medium effect size^45^ in favor of active stimulation.

##### Inhibitory stimulation protocols

Five studies with a total of 84 patients in the experimental group and 79 patients in the sham stimulation group investigated the effects of inhibitory stimulation on PTSD symptoms and all of them applied LF rTMS on the right DLPFC^37,44,46,49,50^. Individual effect estimates showed low heterogeneity (*I*^2^ = 3.85%). Meta-analysis showed a significant positive effect for active compared to sham or no stimulation with a medium effect size (Hedges’ *g* = −0.680) see Fig. 3b. This result was robust to leave-one-out sensitivity analysis (Hedges’ *g* from −1.039 to −0.322). One non-RCT study on the effects of inhibitory rTMS of the right DLPFC, which was not included in our meta-analysis, indicated a positive effect of stimulation on hyperarousal^39^.

##### Studies comparing high versus low frequency stimulation protocols

**Fig. 4:**
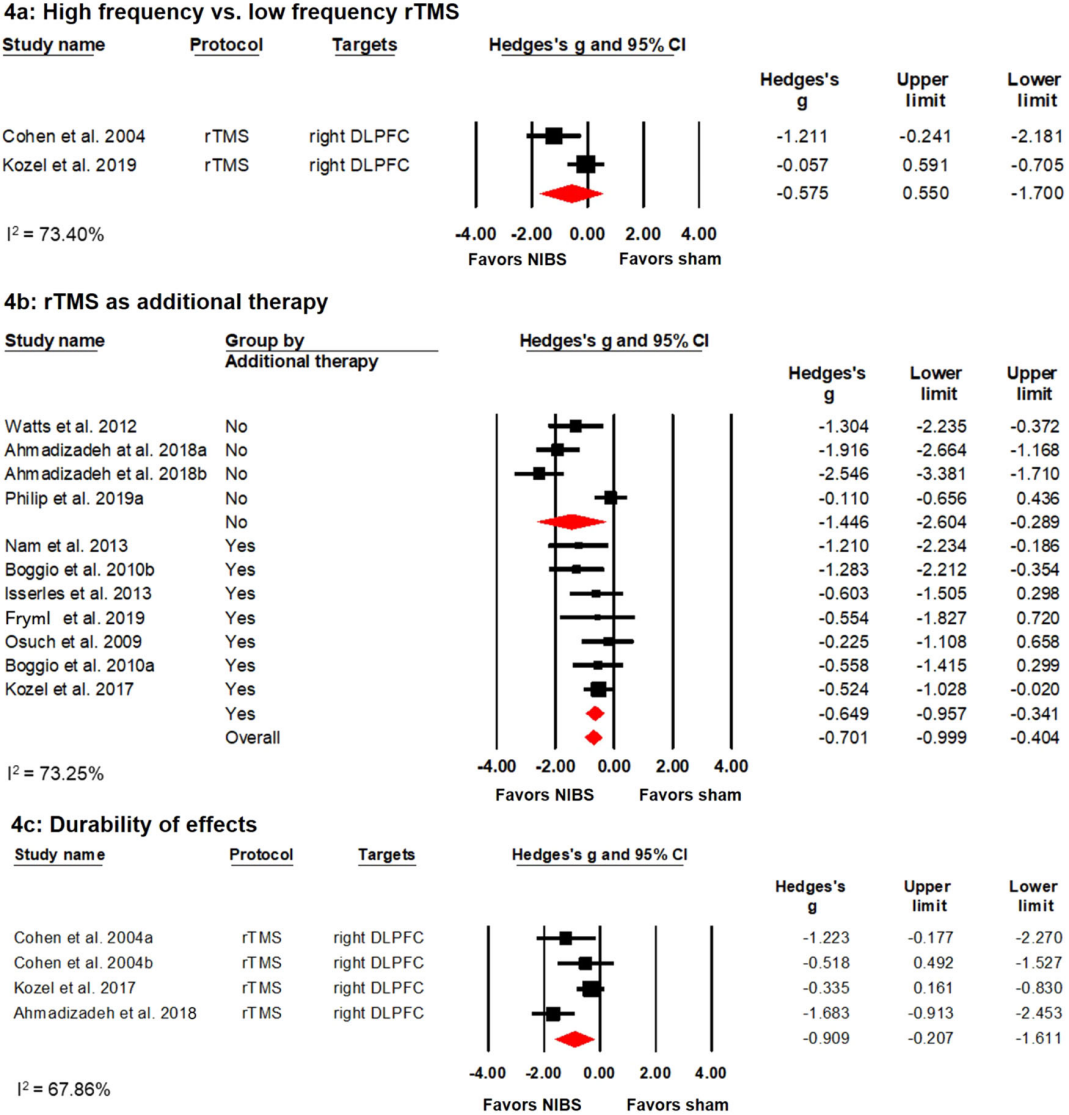
Effects of different NIBS protocols in PTSD. **a** Forest plot showing studies that compared high frequency with low frequency rTMS. **b** Forest plot showing studies using rTMS as an augmentation therapy or not. **c** Forest plot showing studies that investigated the effects of NIBS during follow-up examinations.

In addition, three RCTs investigated the difference between high and low frequency rTMS of the right DLPFC^26,38,46^ with a total of 34 patients in the high frequency stimulation group and 31 in the low frequency group. However, only two studies^38,46^ were suitable for meta-analysis. Studies showed that both high and low frequency rTMS led to significant symptom improvements, while no significant difference was found between the two modes of stimulation, see Fig. 4a).

##### rTMS as an augmentation therapy

Seven datasets from six studies investigated the augmentation effects of rTMS. That is, patients in these studies were allowed to maintain their current psychopharmacological and/or psychotherapeutic treatment during the study period^36,37,45,47,49,50^. Conversely, four datasets from three studies investigated rTMS as monotherapy^35,41,44^. Meta-regression revealed no significant difference (*p* = 0.149) between studies investigating rTMS as mono- or as augmentation therapy. Separate meta-analysis for the two groups indicated that rTMS as well as augmentation therapy showed significant positive effects with a medium (Hedges’ *g* = −0.649) and large (Hedges’ *g* = −1.446) effect size, respectively. (see Fig. 4b).

#### Follow-up

Nine studies explored the durability of NIBS on PTSD, two of them investigating tDCS and the rest applying rTMS stimulation. However, only four data sets (three studies) were suitable for meta-analysis^22,37,46^. Follow-up assessments ranged from two to four weeks. Results indicated durability of effects with large effect size (−0.909) and moderate heterogeneity (*I*^2^ = 67.86%) (see Fig. 4c). This result was robust to leave-one-out sensitivity analysis (Hedges’ *g* from −1.611 to −0.207).

## Discussion

This systematic review including 20 studies, of which 14 datasets were subjected to meta-analysis, revealed significant positive effects of rTMS on the reduction of core PTSD symptoms in patients with PTSD. Subgroup analysis revealed that HF as well as LF rTMS of the right DLPFC is an effective treatment for PTSD with potential durability. Moreover, rTMS seems to be effective as an augmentation treatment for military-related PTSD. However, no dose dependency was revealed in our meta-regression. No definite conclusion could be reached regarding the effects of rTMS on the left DLPFC or MPFC. This may be due to the limited sample size of included studies. The two studies investigating the effects of active compared to sham tDCS both suggested a significant reduction in PTSD symptom with the anode and cathode being placed over the left and the right DLPFC, respectively^22,23^. However, more studies are needed in the future for further quantitative analysis.

Previous evidence showed that activation of the right hemisphere is associated with anxious arousal and symptoms of PTSD during the processing of trauma-specific information^51^. For example, a study measuring regional cerebral blood flow indicated increased blood flow in the right compared to the left hemisphere upon auditory recall of the traumatic event^52^. Our review demonstrates that rTMS targeting the right DLPFC in people with PTSD shows positive effects, which is consistent with previous reviews^18,20^ and in line with a stress modulating effect of right-hemispheric DLPFC stimulation. Interestingly, however, our review indicates that both HF and LF rTMS exerts positive, PTSD symptom reducing effects. A possible reason might lie in the variety of core symptoms of PTSD. Different neural networks and their activity imbalances may underlie the four symptom clusters mentioned in the introduction^21^. More specifically, alterations within and between networks including the default mode network (DMN), the salience network (SN) and the central executive network (CEN), have been associated with PTSD^53^. Reduced functional connectivity within the DMN has been consistently observed and a disorganization between regions belonging to the DMN has been related to the consolidation of trauma-related memories and the preparation for avoidance of trauma reminders. On the other hand, functional connectivity within the SN seems to be increased and a relative SN predominance over DMN has been proposed^53^. Indeed, increased connectivity between DMN and regions belonging to SN and CEN, especially between amygdala and hippocampus, and a decreased connectivity between amygdala and medial prefrontal cortex, were shown to be related to memory intrusion and the re-experiencing of traumatic events^53,54^. The reduced functional connection between the latter two regions has also been linked to excessive fear^53^, whereas a hyperactivation of the right prefrontal cortex and insula, as well as a general neural sensitization has been related to hyperarousal^21,55^. Therefore, it is possible that by influencing different neural networks and associated symptom clusters, both the excitatory and inhibitory stimulation would result in an overall positive effect. This is in line with studies comparing excitatory and inhibitory DLPFC stimulation directly^26,38,46^. Although one study suggested that high-frequency stimulation is superior over low-frequency stimulation, no such effects were found when combining all three studies in our meta-analysis. However, in order to keep side effects such as headache at a minimum, which tend to be stronger for excitatory compared to inhibitory stimulation, clinicians may opt for LF rTMS of the right DLPFC for clinical practice. In any case, well-powered future studies investigating the effects of different rTMS protocols on different symptom clusters in PTSD are needed for definitive answers.

Moreover, several lines of evidence indicate that right stimulation is related to greater improvements in core PTSD symptoms, while left stimulation leads to improvements in mood but only to modest improvements in core trauma symptoms^21,26^. This is consistent with the notion that PTSD is associated with a right-sided pathology and concurs with a study by Cirillo et al., demonstrating the superiority of right prefrontal rTMS to reduce anxiety and PTSD symptoms^20^. The relative severity of symptoms in patients with comorbid PTSD and MDD should therefore determine the decision for applying a left or right stimulation protocol.

In addition, our sub-group analysis examining the augmentation effects of rTMS showed that both mono-, as well as augmentation therapy yielded a significant positive effect, although effect sizes were smaller for augmentation therapy when compared with a control group. This might be due to patients in the control group benefiting from psychopharmacological and/or psychotherapeutic treatment.

Our analysis of studies investigating the durability at follow-up visits indicates positive treatment effects with a durability of at least two to four weeks. This concurs with a recent study not included in our meta-analysis, which explored the long-term effects of iTBS for PTSD^56^. Authors found a clinically meaningful improvement of PTSD symptoms upon iTBS even after a year of treatment. Hence, brain stimulation seems to be a potential alternative approach for the treatment of PTSD, given that two-thirds of patients continue to meet full criteria of PTSD after pharmacological and psychotherapeutic interventions^57^.

## Limitation

There are several limitations in our review. First, we used two different outcome measures in our meta-analysis, the CAPS and the PCL, which is an observer rating and a self-report scale, respectively. Although we computed the standardized mean difference for each outcome, the choice of a specific rating scale may be confounded by the application of a specific stimulation protocol. This was, however, not systematically evaluated in our study. Second, only the total score of CAPS or PCL scales was used for analysis in our review. Hence, a more detailed evaluation of the effects of NIBS on the four main symptom clusters of PTSD in relation to the function of different brain areas awaits to be determined. Third, patient-specific features such as treatment resistance to other therapies may have affected our results. For example, five studies included patients showing a lack of response to an antidepressant medication and/or trauma-focused psychotherapy^22,42,44,47,50^. Future reviews with a sufficient number of studies should investigate this systematically.

## Conclusion

High- as well as low-frequency rTMS of the right DLPFC appears to significantly reduce core PTSD symptoms in patients with PTSD. rTMS may therefore be a promising alternative or add-on treatment for PTSD patients who show limited response to antidepressant medication and/or trauma-focused psychotherapy. More high-quality studies are necessary to explore the effects of NIBS on different symptom clusters in PTSD.

## Supplementary information

Supplement 1
