# Supplementary material for: Non-invasive brain stimulation for posttraumatic stress disorder: a systematic review and meta-analysis
Source: Transl Psychiatry. 2020 May 28;10:168. doi: 10.1038/s41398-020-0851-5 (PMC7256039; doi:10.1038/s41398-020-0851-5)
Supplement: Supplementary file 1 — Supplement 1 [file 41398_2020_851_MOESM1_ESM.docx]

Supplementary Material for

**Non-invasive Brain Stimulation for Posttraumatic Stress Disorder: a systematic review and meta-analysis**

Rebecca L.D. Kan, BSc^1^*, Bella B.B. Zhang, MSc^1^*, Jack J.Q. Zhang, MSc^1^,

Georg S. Kranz, PhD, PD^1,2,3^

*contributed equally

^1^Department of Rehabilitation Sciences, The Hong Kong Polytechnic University, Hong Kong, SAR, China

^2^Department of Psychiatry and Psychotherapy, Medical University of Vienna, Vienna, Austria

^3^The State Key Laboratory of Brain and Cognitive Sciences, The University of Hong Kong, Hong Kong, SAR, China

Table S1. Assessment of the methodological quality of RCTs using the PEDro Scale*

| **Study** | **Eligibility criteria** | **Random allocation** | **Concealed allocation** | **Baseline comparability** | **Blind subjects** | **Blind therapists** | **Blind assessors** | **Adequate follow-up** | **Intention-to-treat analysis** | **Between group comparisons** | **Point estimates and**  **variability** | **Total scores** |
| --- | --- | --- | --- | --- | --- | --- | --- | --- | --- | --- | --- | --- |
| Ahmadizadeh et al. 2018^35^ | Yes | 1 | 1 | 1 | 0 | 0 | 1 | 1 | 1 | 1 | 1 | 8 |
| Boggio et al. 2010^45^ | Yes | 1 | 1 | 1 | 1 | 0 | 1 | 0 | 1 | 1 | 1 | 8 |
| Cohen et al. 2004^46^ | Yes | 1 | 0 | 1 | 1 | 0 | 1 | 1 | 1 | 1 | 1 | 8 |
| Fryml et al. 2019^36^ | Yes | 1 | 1 | 0 | 1 | 0 | 1 | 1 | 1 | 1 | 1 | 8 |
| Isserles et al. 2013^47^ | Yes | 1 | 0 | 1 | 1 | 0 | 1 | 1 | 0 | 1 | 1 | 7 |
| Kozel et al. 2018^37^ | Yes | 1 | 1 | 1 | 1 | 0 | 1 | 0 | 1 | 1 | 1 | 8 |
| Kozel et al. 2019^38^ | Yes | 1 | 1 | 1 | 0 | 0 | 1 | 1 | 1 | 1 | 1 | 8 |
| Nam et al. 2013^49^ | Yes | 1 | 0 | 1 | 1 | 0 | 1 | 1 | 0 | 1 | 1 | 7 |
| Osuch et al. 2009^50^ | Yes | 1 | 0 | 1 | 1 | 0 | 1 | 1 | 1 | 1 | 1 | 8 |
| Philip et al. 2019 a^40^ | Yes | 1 | 0 | 1 | 1 | 0 | 0 | 0 | 1 | 1 | 1 | 6 |
| Philip et al. 2019^41^ | Yes | 1 | 0 | 1 | 1 | 0 | 1 | 1 | 1 | 1 | 1 | 8 |
| Rosenberg et al. 2002^26^ | Yes | 1 | 0 | 1 | 1 | 0 | 1 | 1 | 1 | 1 | 1 | 8 |
| Watts et al. 2012^44^ | Yes | 1 | 0 | 1 | 1 | 0 | 1 | 1 | 1 | 1 | 1 | 8 |
| Ahmadizadeh et al. 2019^22^ | Yes | 1 | 0 | 1 | 1 | 0 | 1 | 1 | 1 | 1 | 1 | 8 |
| Wout et al. 2019^23^ | Yes | 1 | 0 | 1 | 1 | 0 | 0 | 1 | 1 | 1 | 1 | 7 |

0, indicates the criterion was not satisfied; 1, indicates the criterion was satisfied.
